# PSD-95 deficiency disrupts PFC-associated function and behavior during neurodevelopment

**DOI:** 10.1038/s41598-019-45971-w

**Published:** 2019-07-01

**Authors:** Austin A. Coley, Wen-Jun Gao

**Affiliations:** Department of Neurobiology and Anatomy, Drexel University College of Medicine, Philadelphia, PA 19129 USA

**Keywords:** Proteins, Cellular neuroscience, Schizophrenia

## Abstract

Postsynaptic density protein-95 (PSD-95) is a major regulator in the maturation of excitatory synapses by interacting and trafficking N-methyl-D-aspartic acid receptors (NMDAR) and α-amino-3-hydroxy-5-methyl-4-isox-azoleproprionic acid receptors (AMPAR) to the postsynaptic membrane. PSD-95 disruption has recently been associated with neuropsychiatric disorders such as schizophrenia and autism. However, the effects of PSD-95 deficiency on the prefrontal cortex (PFC)-associated functions, including cognition, working memory, and sociability, has yet to be investigated. Using a PSD-95 knockout mouse model (PSD-95^−/−^), we examined how PSD-95 deficiency affects NMDAR and AMPAR expression and function in the medial prefrontal cortex (mPFC) during juvenile and adolescent periods of development. We found significant increases in total protein levels of NMDAR subunits GluN1, and GluN2B, accompanied by decreases in AMPAR subunit GluA1 during adolescence. Correspondingly, there is a significant increase in NMDAR/AMPAR-mediated current amplitude ratio that progresses from juvenile-to-adolescence. Behaviorally, PSD-95^−/−^ mice exhibit a lack of sociability, as well as learning and working memory deficits. Together, our data indicate that PSD-95 deficiency disrupts mPFC synaptic function and related behavior at a critical age of development. This study highlights the importance of PSD-95 during neurodevelopment in the mPFC and its potential link in the pathogenesis associated with schizophrenia and/or autism.

## Introduction

Postsynaptic density-95 (PSD-95), a member of the membrane-associated guanylate kinase family (MAGUK)^1^, is a major scaffolding protein located within the postsynaptic density (PSD) of excitatory synapses. PSD-95 is involved in the recruitment, trafficking and stabilization of N-methyl-D-aspartic acid receptors (NMDARs) and α-amino-3-hydroxy-5-methyl-4-isox-azoleproprionic acid receptors (AMPARs) to the postsynaptic membrane during cortical development^2,3^. PSD-95 is an essential component involved in glutamatergic transmission, synaptic plasticity, and dendritic spine morphogenesis during neurodevelopment^2–4^. Recently, genomic studies link PSD-95 dysfunction to neuropsychiatric disorders such as schizophrenia (SCZ), autism spectrum disorder (ASD), and intellectual disorder (ID)^5–7^; however, the underlying mechanisms have yet to be elucidated.

PSD-95 plays a critical role in synaptic plasticity of glutamatergic synapses due to its interaction and functional implications of NMDA and AMPA receptors. For instance, PSD-95 binds directly to carboxy-terminal tails of NMDA receptor subunits, GluN2A and GluN2B, via PDZ domains; and indirectly binds AMPA receptors through stargazin/TARPs interaction^8^. A previous study performed within the hippocampus revealed PSD-95 deficiency causes a significant decrease in AMPAR-mediated current, suggesting silent synapse formation^9^. Similar observations were shown in the V1 cortex describing PSD-95 as a regulator of silent synapse formation and mediator of ocular dominant plasticity^10^. Further evidence within the hippocampus describes PSD-95 along with other MAGUK scaffolding proteins such as SAP-102 and PSD-93, as key contributors to NMDAR expression and function^11,12^. For instance, PSD-95 knockdown studies show an increase in GluN2B clustering at dendritic spines^13^, and a triple knockdown of PSD-95/SAP-102/PSD-93 attenuates NMDAR current^12^. These studies infer PSD-95 deficiency causes an imbalance of NMDAR and AMPAR presence and function, thereby altering glutamatergic transmission.

Synaptic dysregulation at the dendritic spines within SCZ and ASD patients have been localized to cortical regions such as the medial prefrontal cortex (mPFC), a region responsible for cognition, working memory and sociability^14,15^. More specifically, PSD-95 mRNA and protein expression levels are downregulated in the mPFC of SCZ patients^16,17^. Therefore, it is plausible that PSD-95 disruption within the mPFC could lead to the cognitive and social impairments associated with neuropsychiatric disorders. However, the molecular and cellular effects in response to PSD-95 deficiency on the mPFC function have yet to be elucidated. We hypothesized that PSD-95 deficiency (Supplemental Figs 1 and 2) will disrupt synaptic maturation during critical periods of neurodevelopment due to an alteration in NMDAR/AMPAR-glutamatergic transmission that leads to impairments in mPFC development and function. By utilizing a PSD-95 knockout mouse model, this study characterizes changes in specific NMDAR-and AMPAR-subunit expression levels at the synapse, accompanied with fluctuations in SAP-102 and PSD-93 protein levels in response to PSD-95 deficiency in the mPFC. We also examine the effects of PSD-95 deficiency on synaptic function in an age-dependent manner during development, thus enabling us to identify a critical period at which PSD-95 deficiency disrupts synaptic maturation in the mPFC. Additionally, we assess mPFC synaptic function by recording NMDAR/AMPAR current and short-term plasticity measurements using whole-cell patch clamp recordings. And lastly, we evaluate mPFC-associated behavior such as cognition, working memory and sociability. This study provides great insight into the effects of PSD-95 deficiency on synaptic function and development of the mPFC and its potential implications in aberrant behavior associated with neuropsychiatric disorders.

## Results

### AMPAR and NMDAR protein levels are altered during a critical period of development in the mPFC of PSD-95^−/−^ mice

**Figure 1:**
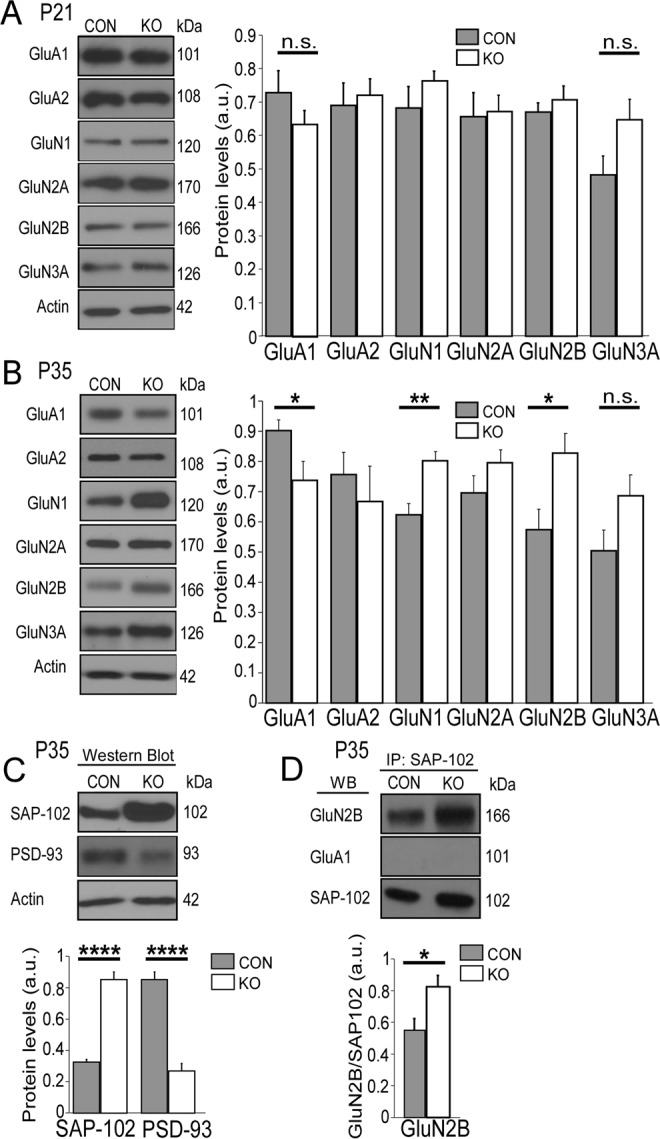
PSD-95 deficiency alters specific AMPAR and NMDAR-subunit protein expression levels in the mPFC at postnatal day 35, but not postnatal day 21. (**A**) Left panel shows representative blots for AMPAR subunits (GluA1 & GluA2) and NMDAR subunits (GluN1, GluN2A, GluN2B, and GluN3A) in control (Con) versus PSD-95 knockout (KO) mice at P21. All proteins were normalized to actin. Right panel displays summary graphs of protein expression levels in arbitrary units (a.u.) of GluA1 (p = 0.28, Con, n = 7, KO, n = 8), GluA2 (p = 0.39, n = 6), GluN1 (p = 0.23, Con, n = 7, KO, n = 10), GluN2A (p = 0.85, Con, n = 8, KO, n = 10), GluN2B (p = 0.51, Con, n = 7, KO, n = 10), GluN3A (p = 0.15, Con, n = 8, KO, n = 9) in Con vs. KO mice. (**B**) Left panel shows representative blots for AMPAR subunits, NMDAR subunits and actin at P35 in con vs. KO mice. Right panel displays summary graphs of protein expression levels in a.u. in GluA1 (p = 0.04, n = 7), GluA2 (p = 0.51, Con, n = 8, KO, n = 5) GluN1 (p = 0.002, Con, n = 10, KO, n = 8); GluN2A (p = 0.18, Con, n = 9, KO, n = 8) GluN2B (p = 0.02, n = 7), GluN3A (p = 0.08, Con, n = 9, KO, n = 8) in Con vs. KO mice. PSD-95 deficiency causes a significant increase in SAP-102 that leads to an increase in interaction of GluN2B in the mPFC. (**C**) Upper panel shows representative western blots of SAP-102 and PSD-93 in control versus PSD-95 knockout mice. Both proteins were normalized to actin. Lower panel displays summary graphs of protein expression levels of SAP-102 and PSD-93 in Con vs. KO mice (SAP-102, p = 9 × 10^−7^, n = 7; PSD-93, p = 3 × 10^−6^, Con, n = 7, KO, n = 6). (**D**) Upper panel shows blots of SAP-102 and GluN2B co-immunoprecipitation in Con vs. KO mice. GluA1 was used as a negative control. Lower panel displays a summary graph of protein levels of GluN2B and SAP-102 interaction (GluN2B/SAP-102, p = 0.03, n = 5). All full length/uncropped western blots are presented in Supplementary Fig. 2. *p < 0.05, **p < 0.01, ****p < 0.0001, n.s., not significant.

To determine if AMPAR and NMDAR protein levels are altered during development in the mPFC of PSD-95 deficient mice, we used western blot analysis to examine specific AMPAR subunits GluA1 and GluA2; and NMDAR subunits GluN1, GluN2A, GluN2B and GluN3A protein expression levels at juvenile (P21) and adolescent (P35) age ranges. Our results revealed no changes in either AMPAR subunits or NMDAR subunits in juvenile PSD-95^−/−^ mice compared to control mice (GluA1, Con, 0.73 ± 0.06, KO, 0.64 ± 0.04, p = 0.28; GluA2, Con, 0.72 ± 0.04, KO, 62 ± 0.08, p = 0.39; GluN1, Con, 0.68 ± 0.07, KO, 0.76 ± 0.03, p = 0.23; GluN2A, Con = 0.66 ± 0.07, KO = 0.67 ± 0.05, p = 0.85; GluN2B, Con, 0.67 ± 0.03, KO, 0.71 ± 0.04, p = 0.51; GluN3A, Con, 0.48 ± 0.06, KO, 0.61 ± 0.05, p = 0.15; Fig. 1A). However, during the adolescent age range we observed a significant decrease in GluA1 within PSD-95^−/−^ mice compared to the control (GluA1, Con, 0.90 ± 0.04, KO, 0.73 ± 0.06, p = 0.04; Fig. 1B). Interestingly, our results also showed significant increases in NMDAR-subunits GluN1 and GluN2B in PSD-95^−/−^ mice during adolescence (GluN1, Con, 0.62 ± 0.04, KO, 0.80 ± 0.03, p = 0.002; GluN2B, Con, 0.57 ± 0.07, KO, 0.83 ± 0.07, p = 0.02; Fig. 1B). No changes were observed in GluA2 or GluN2A (GluA2, Con, 0.76 ± 0.07, KO, 0.67 ± 0.10, p = 0.51; GluN2A, Con, 0.70 ± 0.06, KO = 0.80 ± 0.04, p = 0.18); however, we noticed a slight increase but not statistically significant change in GluN3A within PSD-95^−/−^ mice (GluN3A, Con, 0.50 ± 0.07, KO, 0.68 ± 0.07, p = 0.08). These results indicate PSD-95 deficiency alters specific NMDAR-and AMPAR-subunit protein expression levels in the mPFC at a critical period during development.

**Figure 2:**
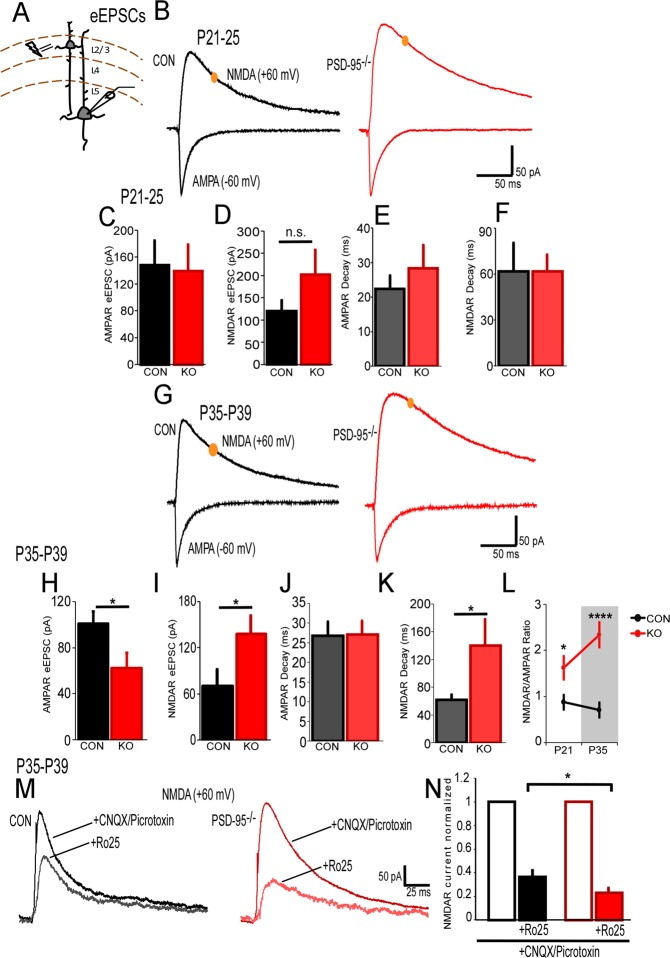
PSD-95 deficiency causes an increase in NMDAR/AMPAR-current ratio during development in layer V pyramidal neurons. (**A**) Schematic of layer II/III stimulation and layer V whole-cell patch clamp recordings of pyramidal neurons in the mPFC. (**B**) Example traces of AMPAR eEPSCs recorded at −60 mV and NMDAR eEPSCs recorded at +60 mV in the presence of picrotoxin from control and PSD-95^−/−^mice at postnatal days 21–25. NMDAR eEPSC analysis was measured 50-ms post-stimulus as illustrated by the yellow circle. (**C** & **D**) Graphs show mean peak amplitudes of AMPAR-eEPSCs (p = 0.77, n = 10) and NMDAR-eEPSCs (p = 0.31, n = 10) at P21-25. (**E**,**F**) Graphs represent AMPAR and NMDAR decay time constant (τ) ± s.e.m. in ms (AMPAR, p = 0.31; NMDAR, p = 0.44, n = 10). (**G**) Example traces of AMPAR eEPSCs recorded at −60 mV and NMDAR eEPSCs recorded at +60 mV in the presence of picrotoxin from control and PSD-95^−/−^mice at postnatal days 35–39. (**H**,**I**) Graphs show mean peak amplitudes of AMPAR-eEPSCs and NMDAR-eEPSCs (AMPAR, p = 0.03; NMDAR; p = 0.03, Con, n = 16, KO, n = 14). (**J** & **K**) Graphs represent AMPAR and NMDAR decay time constant (AMPAR, p = 0.96; NMDAR, p = 0.04 Con, n = 16, KO, n = 14). (**L**) Summary of mean NMDAR/AMPAR-current ratio ± s.e.m, ANOVA, F(1,46) = 23.457,p = 1 × 10^−5^; paired *t* tests, P21-25, p = 0.02; P35-P39, p = 7 × 10^−5^, Con, n = 16, KO, n = 14. (**M**) Example traces of NMDAR eEPSCs recorded at +60 mV in the presence of CNQX and picrotoxin from control and PSD-95^−/−^mice at postnatal day 35–39. Ro25 was then bath applied to block GluN2B-containing NMDARs. (**N**) Quantifications show normalized NMDAR-eEPSCs (p = 0.043, Con, n = 7, KO, n = 8). *p < 0.05, ****p < 0.0001, n.s., not significant.

Since we detected an increase in NMDAR subunits GluN1 and GluN2B in PSD-95^−/−^ mice within the mPFC, we investigated other potential scaffolding proteins that may overcompensate in the recruitment and/or stabilization of NMDAR’s within the PSD. Therefore, using western blot analysis we targeted SAP-102 and PSD-93, which are known to bind to GluN2 specific subunits^18–20^. Our results revealed a dramatic increase in SAP-102 protein expression levels during adolescence in PSD-95^−/−^ mice compared to control mice (SAP-102, Con, 0.32 ± 0.03, KO, 0.85 ± 0.05, p < 0.0001; Fig. 1C). And interestingly, we observed a significant reduction in PSD-93 protein expression levels in PSD-95^−/−^ mice (PSD-93, Con, 0.85 ± 0.05, KO, 0.27 ± 0.05, p < 0.0001). Furthermore, we utilized co-immunoprecipitation to measure the interaction between GluN2B and SAP-102 within the PSD. Our results revealed a significant increase in the interaction of GluN2B and SAP-102 in PSD-95^−/−^ mice compared to control mice in the mPFC (GluN2B/SAP-102, Con, 0.55 ± 0.07, KO, 0.82 ± 0.07, p = 0.03; Fig. 1D). Therefore, this data indicate that PSD-95 deficiency promotes an upregulation in SAP-102 protein expression levels that lead to a significant interaction with GluN2B, and thus recruitment and/or stabilization of GluN2B-NMDARs within the PSD.

### NMDAR/AMPAR-mediated current increases during development in mPFC layer V pyramidal neurons in PSD-95^−/−^ mice

To examine NMDAR-and AMPAR evoked excitatory postsynaptic currents (eEPSCs) in the PSD-95 deficient mice, we stimulated layer II/III and recorded from layer V pyramidal neurons in the mPFC (Fig. 2A,B). Our results revealed no differences in peak amplitudes or decay times of AMPAR-eEPSCs, and NMDAR-eEPSCs in juvenile PSD-95^−/−^ mice compared to control mice (AMPAR-eEPSC pA, Con, 148.38 ± 35.91, KO, 139.14 ± 39.46, p = 0.77; AMPAR-eEPSC decay ms, Con, τ = 22.38 ± 3.86, KO, τ = 28.33 ± 6.75, p = 0.31, Fig. 2C,E; NMDAR-eEPSC pA, Con, 120.31 ± 23.73, KO, 202.02 ± 55.40, p = 0.16; NMDAR-eEPSC decay ms, Con, τ = 61.73 ± 18.49 m.s., KO, τ = 73.56 ± 10.98 m.s., p = 0.44; Fig. 2D,F). However, we observed a significant decrease in the peak amplitude of AMPAR-eEPSCs, accompanied with a significant increase in NMDAR-eEPSCs in adolescent PSD-95^−/−^ mice compared to control mice (AMPAR-eEPSC pA, Con, 101.07 ± 10.56, KO, 62.55 ± 12.85, p = 0.03; NMDAR-eEPSC pA, Con, 70.46 ± 21.05, KO, 138.04 ± 23.49, p = 0.03, Fig. 2H,I). Interestingly, there was an increase in NMDAR-EPSC decay time in adolescent PSD-95^−/−^ mice compared to the controls (NMDAR-eEPSC decay ms, Con, τ = 61.75 ± 7.6 m.s., KO, τ = 140.16 ± 37.46 m.s., p = 0.04; Fig. 2K), suggesting an increase in GluN2B-containing NMDARs due to a longer open probability of the receptor channel. No differences were observed in AMPAR-eEPSC decay time (AMPAR-eEPSC decay ms, Con, τ = 26.79 ± 3.53, KO, τ = 27.07 ± 3.43, p = 0.96, Fig. 2J). Furthermore, we calculated the NMDAR/AMPAR-mediated current ratio in juvenile and adolescent age ranges, and our results revealed significant increases in NMDAR/AMPAR-current ratio that progress during development (P21-25, Con, 0.87 ± 0.16, KO, 1.62 ± 0.26, p = 0.02; P35-P39, Con, 0.79 ± 0.16, KO, 2.21 ± 0.27, p < 0.0001; Fig. 2L). In addition, to further corroborate the presence and function of GluN2B-containing NMDARs in adolescent PSD-95^−/−^ mice, we applied Ro25-6981 (Ro25), a selective GluN2B antagonist. Ro25 blocked 79.6% ± 3.8% of NMDAR current in PSD-95^−/−^ mice, compared to 63.5% ± 5.7% in control mice (Fig. 2M,N), thereby indicating GluN2B-containing NMDARs are dominant in layer 5 pyramidal neurons in PSD-95 deficient mice.

**Figure 3:**
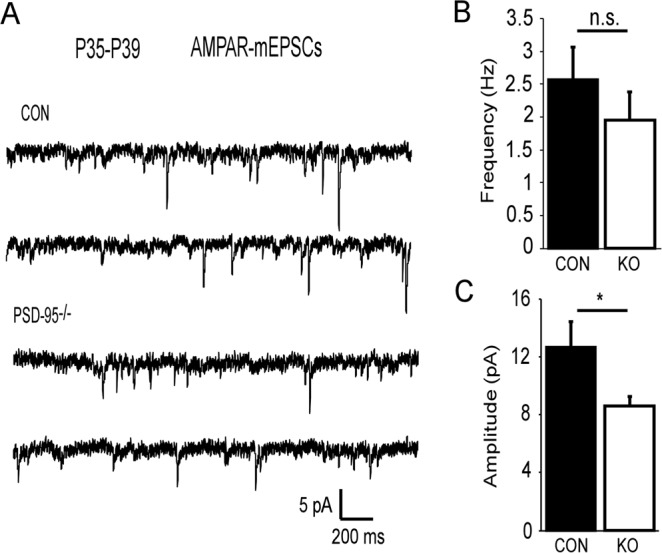
(**A**) Example traces of AMPAR mEPSCs recorded at −70 mV in the presence of picrotoxin and TTX from control and PSD-95^−/−^mice at postnatal days 35–39. (**C**) Summary graphs of mEPSC frequency (p = 0.36) and amplitude (p = 0.045, Con, n = 8, KO, n = 9). *p < 0.05, n.s., not significant.

Additionally, we measured AMPAR miniature excitatory postsynaptic currents (mEPSCs) in layer V pyramidal neurons (Fig. 3A). Our results showed no significant changes in the frequency of mEPSCs in PSD-95^−/−^ mice compared to control mice (Con, 2.56 ± 0.51 Hz, KO, 1.94 ± 0.43 Hz, p = 0.36; Fig. 3B). However, we observed a significant reduction in the amplitude (Con, 12.64 ± 1.81 pA, KO, 8.58 ± 0.69 pA, p = 0.045; Fig. 3C). These results indicate the decrease in AMPAR transmission is predominately due to a reduction in the postsynaptic response. Furthermore, we investigated action potential firing and the membrane properties of layer V pyramidal neurons in PSD-95^−/−^ mice. We showed no significant differences in spike numbers in PSD-95^−/−^ mice compared to control mice (F = 2.47, p = 0.13; Supplemental Fig. 3A,B). However, we revealed a significant increase in the rheobase in PSD-95^−/−^ mice compared to the control (Con, 90 ± 12.15, KO, 143.33 ± 14.53, p = 0.008; Supplemental Table 1), thereby indicating these neurons require a high current injection to induce an action potential. We observed no differences in resting membrane potential, input resistance, action potential peak amplitude, action potential spike threshold, and action potential ½ width (Supplemental Table 1). These results suggest that action potentials and membrane properties in layer V pyramidal neurons are not altered in response to PSD-95 deficiency.

### Excitatory postsynaptic potentials are reduced but temporal summation remains normal in mPFC layer V pyramidal neurons in PSD-95^−/−^ mice

**Figure 4:**
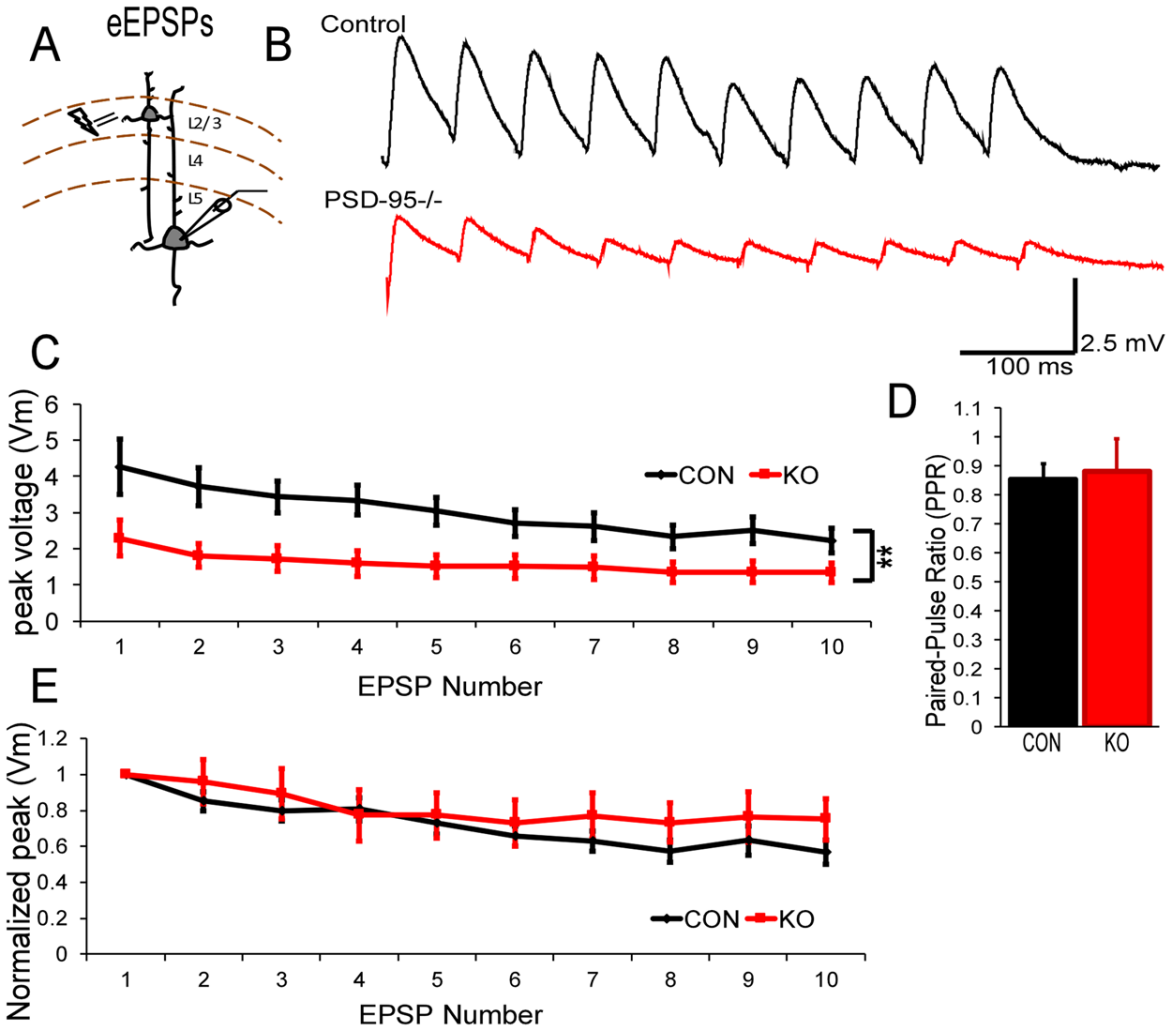
PSD-95 deficiency attenuates eEPSPs and normal temporal summation in layer V pyramidal neurons in the mPFC. (**A**) Schematic of layer II/III stimulation and whole-cell patch clamp recordings of layer V pyramidal neurons in the mPFC. (**B**) Representative traces of 10-pulse AMPAR-eEPSPs recorded at −70 mV from control and PSD-95^−/−^mice at postnatal day 35. (**C**) Summary graph of peak voltage (Vm) of eEPSPs (Repeated measures of ANOVA; F(1,26) = 8.23, p = 0.008, Con, n = 12, KO, n = 14). (**D**) Paired-pulse ratio (PPR) was calculated from the measured peak 2 Vm /peak 1 Vm (p = 0.42, n.s., Con, n = 12, KO, n = 14). (**E**) Summary graph of normalized peak (Vm) (Repeated measures of ANOVA; F(1, 24) = 0.34, p = 0.86, Con, n = 12, KO, n = 14). **p < 0.01.

Short-term plasticity is a critical component in the mPFC as it relates to cognitive processes such as working memory^21^. Therefore, we investigated the role of PSD-95 deficiency by eliciting a 10-pulse stimulation within layer II/III and recording AMPAR-meditated excitatory postsynaptic potentials (eEPSPs) in layer V pyramidal neurons in the mPFC (Fig. 4A). We found a significant reduction in AMPAR-eEPSPs in PSD-95^−/−^ mice compared to control mice (Repeated measures ANOVA; group: F = 8.23, p = 0.008; Fig. 4B,C). However, we found no differences in the paired-pulse ratio, suggesting no changes in presynaptic release (Con, 0.85 ± 0.05, KO, 0.88 ± 0.11, p = 0.42; Fig. 4D). In addition, we observed no differences in normalized EPSPs within PSD-95^−/−^ mice (Repeated measures ANOVA; F = 0.34, p = 0.86; Fig. 4E), thereby indicating no changes in temporal summation. Nevertheless, this data suggest that the reduction in AMPAR-eEPSPs is due to a lack of GluA1-containing AMPARs in PSD-95 deficient mice.

### PSD-95^−/−^ mice exhibit sociability deficits

**Figure 5:**
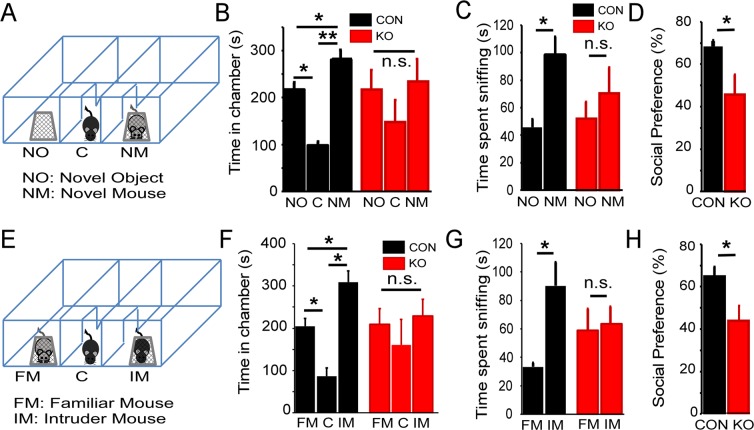
PSD-95^−/−^ mice are socially impaired and display a lack of social novelty and exploration. (**A**) Diagram depicting a subject mouse in the center chamber of a 3-chamber apparatus that includes a novel mouse in one chamber and a novel object in the other chamber comparing PSD-95^−/−^mice and control mice at postnatal day 35. (**B**) Summary graphs show time (seconds) spent in each chamber (novel object, center, novel mouse). The time spent in chamber, ANOVA, F(2,57) = 6.694, p = 0.002. (**C**) The time spent sniffing, ANOVA, F(1,38) = 6.192, p = 0.017 with paired *t*-tests CON, KO, p = 0.347. (**D**) Social preference was calculated by the time spent sniffing stranger mouse divided by total time spent sniffing × 100 (% ± s.e.m.), p = 0.047. (**E**) Diagram representing a subject mouse in the center chamber, and familiar mouse and intruder mouse in side chambers. (**F**) Summary graphs show time (seconds) spent in each chamber (familiar mouse, center, intruder mouse). The time spent in chamber, ANOVA, F(2,57) = 6.556, p = 0.003. (**G**) The time spent sniffing, ANOVA, F(1, 38) = 3.413, p = 0.012; paired *t*-tests CON, p = 0.033, KO, p = 0.792. (**H**) Social preference was calculated. p = 0.036. *p < 0.05, **p < 0.01, n.s., not significant.

To assess the behavioral attributes of PSD-95^−/−^ mice, we first measured locomotor activity in the novel open field for 30 min. PSD-95^−/−^ mice displayed no difference in locomotor activity compared to control mice, suggesting normal locomotion and/or motor function (Repeated measures ANOVA; F = 0.90, p = 0.37; Supplemental Fig. 4). However, to examine mPFC-associated behavior such as sociability, we utilized a 3-chamber apparatus to test social approach and social novelty/exploration in PSD-95^−/−^ mice at P35. Our results revealed PSD-95^−/−^ mice show a reduced preference in interacting with a novel mouse relative to an object compared to control mice, as measured by the amount of time spent sniffing in each chamber (time spent in chamber, ANOVA, F = 6.694, p = 0.002; time spent sniffing, ANOVA, F = 6.192, p = 0.017; paired *t*-tests CON, p = 0.018, KO, p = 0.347; social preference, CON, 68.43% ± 2.54, KO, 45.67% ± 9.43, p = 0.048; Fig. 5A–D). In the next session—examining a familiar mouse versus an intruder mouse —PSD-95^−/−^ mice showed a reduced social preference for the intruder mouse compared to control mice, indicating a lack of social novelty and exploration (chamber, ANOVA, F = 6.556, p = 0.003; sniffing, ANOVA, F = 3.413, p = 0.012; paired *t*-tests CON, p = 0.033, KO, p = 0.792; social preference, CON, 65.11% ± 4.5, KO, 44.03% ± 7.32, p = 0.036; Fig. 5E–H). This data suggests that PSD-95^−/−^ mice exhibit sociability deficits that are consistent with symptoms of neuropsychiatric patients.

### PSD-95^−/−^ mice exhibit recognition memory and display learning deficits

**Figure 6:**
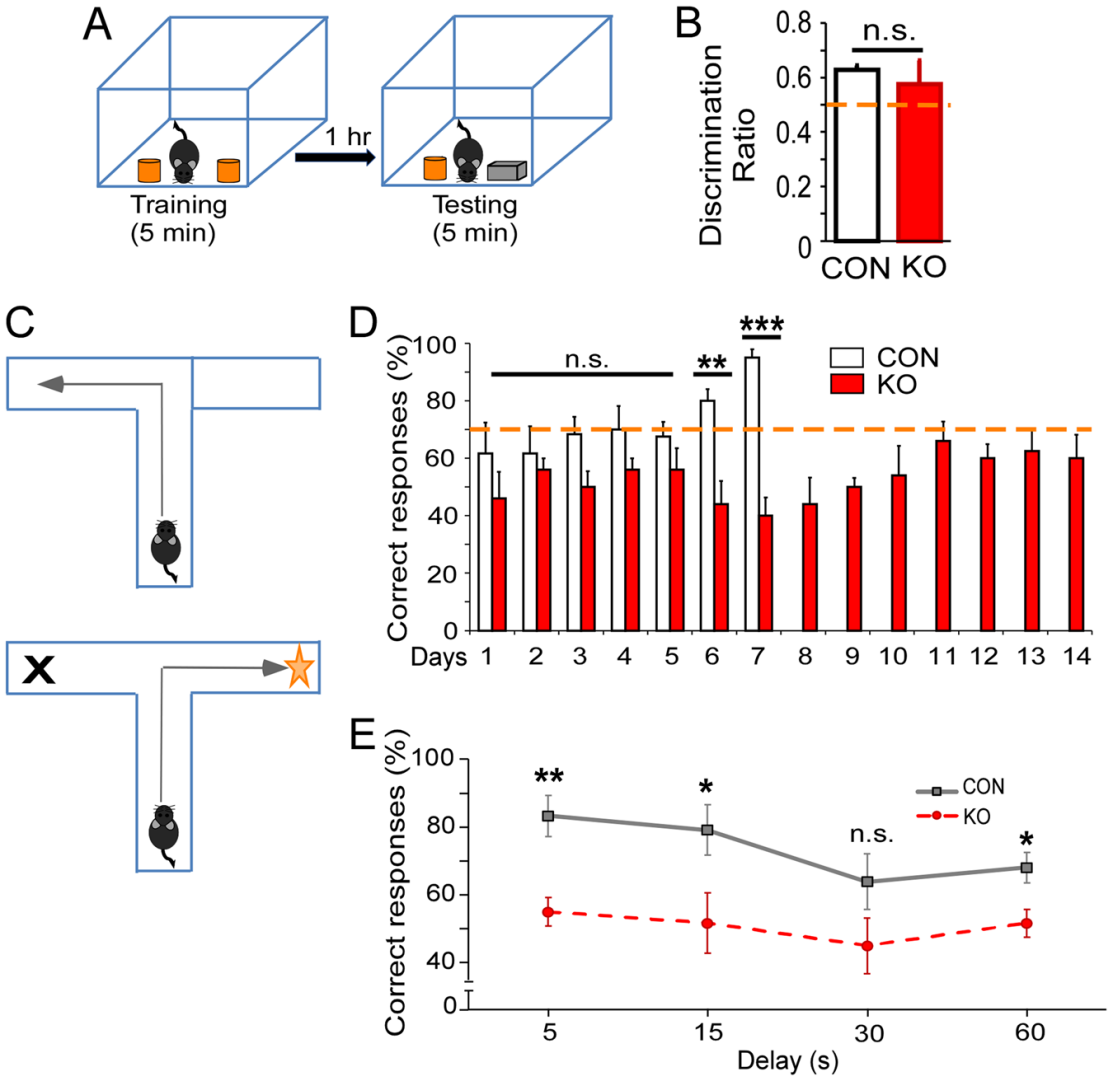
PSD-95^−/−^ mice display normal recognition memory but impaired learning and working memory. (**A**) Schematic showing the subject mouse during novel object recognition task. (**B**) A discrimination ratio (novel object interaction/total interaction with both objects) was calculated to measure time spent sniffing familiar object versus the novel object in PSD-95^−/−^mice versus control mice at postnatal day 35. Control mice differ significantly from 0.5 by one-sample t-test (*t*(7) = 8.140, p = 8 × 10^−5^), but PSD-95^−/−^mice does not differ (*t*(6) = 0.896, p = 0.405). Paired *t-*tests show no significant differences between PSD-95^−/−^mice and control mice (p = 0.54, CON, n = 8, KO, n = 7, n.s.). (**C**) Schematic of the T-maze discrete paired-trial delayed alternation task. (**D**) Days to criterion: (ANOVA, F(1,54) = 25.85, p = 5 × 10^−6^; followed by paired *t*-tests on day 6 and 7 in PSD-95^−/−^mice versus control mice beginning at postnatal day 35 (day 1, p = 0.14, day 2, p = 0.60, day 3, p = 0.09, day 4, p = 0.19, day 5, p = 0.33 day 6, p = 0.006; day 7, p = 0.0003; CON, n = 6, KO, n = 5). (**E**) Working memory task with multiple delay periods (repeated measures ANOVA, F(1,9) = 9.04, p < 0.05, paired *t*-test on 5 sec, p = 0.005, 15 sec, p = 0.039, 30 sec, p = 0.141, 60 sec, p = 0.027. *p < 0.05, **p < 0.01, ***p < 0.0001, n.s., not significant.

To further examine mPFC-associated behaviors, we evaluated cognition and memory in PSD-95^−/−^ mice at P35. In the novel object recognition task, PSD-95^−/−^ mice showed no preferences to the novel object (one sample t-test, *t* = 0.896, p = 0.405), thus displaying recognition memory dysfunction; although, interestingly, showed no significant differences compared to control mice (CON, 0.63 ± 0.016, KO, 0.58 ± 0.086, p = 0.54; Fig. 6A,B). However, when performing the T-maze task, PSD-95^−/−^ mice showed a reduction in correct responses to reach criterion (70% correct responses) compared to control mice (ANOVA, F = 25.85, p < 0.0001; Fig. 6C,D). Furthermore, PSD-95^−/−^ mice performed worse during delay tasks (repeated measures ANOVA, F = 9.04, p = 0.015; paired *t*-tests, 5 sec, CON, 83.3% ± 6.08, KO, 55% ± 4.24, p = 0.005; 15 sec, CON, 79.16% ± 7.38, KO, 51.67% ± 8.90, p = 0.039; 30 sec, CON, 63.89% ± 8.24, KO, 45% ± 8.16, p = 0.141; and 60 sec, CON, 68.06% ± 4.52, KO, 51.67% ± 4.08, p = 0.027; Fig. 6E). Together, these results indicate PSD-95^−/−^ mice display learning and working memory deficits.

## Discussion

The mPFC is a major region within the cortex responsible for executive functions, such as cognition, working memory, and sociability. However, this region is highly susceptible—largely due to synaptic dysregulation—in patients diagnosed with neuropsychiatric disorders, thus contributing to symptoms that include cognitive deficits and sociability impairments^22,23^. PSD-95, a major component responsible for synaptic maturation, has recently been associated with neuropsychiatric disorders^4,24–27^. However, despite the importance of PSD-95 in excitatory synaptic function and impairment in diseases, how PSD-95 deficiency affects PFC-associated function remain unexplored. Previous studies using a PSD-95 knock-out model reveal decreases in AMPAR/NMDAR current ratio in the hippocampus at postnatal day 14, and within the visual cortex at postnatal day 30^9,10^; thus, indicating region-specific developmental changes that occur due to PSD-95 deficiency. Our study, although revealing a similar reduction in the AMPAR/NMDAR current ratio, reports these changes occuring at postnatal day 35 in the mPFC of PSD-95^−/−^ mice.

Our results showed that synaptosomal AMPAR- and NMDAR-subunits protein expression levels are altered during the adolescent, but not juvenile, age range in PSD-95 deficient mice, thus identifying a critical period at which PSD-95 is most susceptible in the mPFC. Accordingly, previous studies have shown that within the normal cortex, PSD-95 proteins levels increase from early life and peak during adolescence/early adulthood^28,29^, suggesting a development age range at which PSD-95 greatly influences synaptic maturation. We revealed a significant reduction in AMPAR-subunit GluA1, accompanied by a significant increase in NMDAR-subunits GluN1 and GluN2B in the absence of PSD-95 during adolescence (but not juvenile). This is contrary to normal synaptic development, as AMPARs demonstrate an increase in recruitment and stabilization compared to NMDARs^30^. We showed that the aberrant increase in NMDAR subunits observed in PSD-95^−/−^ mice was attributed to a compensatory increase in SAP-102 protein expression, and is corroborated with an increase in the interaction of GluN2B-containing NMDAR subunits. Therefore, we provide a novel mechanism describing a pivotal role of SAP-102 in response to PSD-95 deficiency and the dynamic responsibilities of MAGUK scaffolding proteins at the PSD. Interestingly, PSD-93, involved in the formation of heteromulterization complexes of PSD-95 and NMDARs^31^, is dramatically reduced in the mPFC of PSD-95^−/−^ mice. This data suggest a reduction in PSD-93/PSD-95 complexes at the PSD and further describes the dominant role of SAP-102 in the absence of PSD-95 and its effect on NMDARs. NMDARs consist of an obligatory GluN1 subunit and are involved in a GluN2B-to-GluN2A subunit switch that occurs from childhood-to-adulthood^32^ in most cortical regions, whereas GluN2B protein levels remain high in the mPFC, and is thereby important for cognitive processes^33–35^.

Furthermore, SAP-102 preferentially binds GluN2B to form SAP-102/GluN2B complexes in immature synapses and are replaced by PSD-95/GluN2A complexes later in development^36^. Therefore, high expression levels of SAP-102/GluN2B due to PSD-95 deficiency would disrupt normal synaptic maturation within the PFC. GluN2B-containing NMDA receptors have slow kinetics and thus play a major role in calcium (Ca^+^) influx at the postsynaptic membrane; however, an overabundance may result in a significant increase in Ca^+^ conductance that could lead to excitotoxicity and neuronal damage^37^. Therefore, we expect the increase in GluN2B presence due to PSD-95 deficiency would impair mPFC tissue function, thus leading to behavioral deficits.

Our electrophysiology data revealed an increase in the NMDAR/AMPAR-mediated current ratio in layer V pyramidal neurons in PSD-95^−/−^ mice that progresses during development. We showed no differences in AMPAR-eEPSCs or NMDAR-eEPSCs at the juvenile age range (P21-25); and thus, is consistent with the observed AMPAR and NMDAR subunits protein expression levels in the mPFC of PSD-95 deficient mice. However, during the adolescence age range (P35-P39), we observed a significant decrease in AMPAR-eEPSCs, accompanied with an increase in the NMDAR-eEPSCs peak amplitude and decay time, that is indeed consistent with the observed decrease in AMPAR subunits GluA1 and increase in NMDAR GluN1 and GluN2B protein expression levels from western blot analysis. This data describe and corroborate that alterations in AMPAR and NMDAR protein expression levels at the PSD affect AMPA and NMDA-receptor function activity. Additionally, this data characterize the critical adolescent time point during development at which PSD-95 deficiency alters mPFC AMPAR and NMDAR protein expression levels and function.

We examined mPFC-associated behavioral phenotypes that assessed sociability, cognition, and working memory in PSD-95^−/−^ mice. In a previous study, a group characterized behavioral domains of mice with total PSD-95 deletion (*Dlg4*^−/−^), and showed *Dlg4*^−/−^ mice displayed repetitive behavior, cognitive deficits, disrupted motor coordination; however, no differences in unconditioned anxiety behaviors or social interaction^38^. In our study, PSD-95^−/−^ mice revealed no significant differences in locomotor activity, thereby displaying normal spontaneous locomotion compared to control mice. PSD-95^−/−^ mice showed a lack of social exploration and novelty, and thus describe reduced sociability as observed in SCZ patients^39,40^. However, it is plausible that regions other than the mPFC responsible for sociability are susceptible to PSD-95 deficiency, such as the nucleus accumbens (NAc), amygdala, and hypothalamus^41,42^. For instance, it was recently shown that the neuropeptide oxytocin regulates synaptic plasticity in the NAc during critical periods of neurodevelopment^42^. Therefore, a down-regulation of PSD-95 in the NAc during development is likely to disrupt synaptic plasticity that would lead to aberrant sociability. Moreover, the mPFC has a top-down influence on these subcortical regions^41^; thus, PSD-95 deficiency may cause a reduction in mPFC output that would reduce sociability.

We also assessed the recognition memory of PSD-95^−/−^ mice during the novel object recognition task. Recognition memory involves the mPFC and hippocampus, as the task includes memory consolidation and retrieval^43,44^, although some studies suggest the mPFC is not a requirement for simple recognition tasks^45,46^. Interestingly, PSD-95^−/−^ mice showed no differences compared to control mice in discrimination ratio during the tasks, although displayed recognition memory deficits to the novel object. Therefore, since PSD-95^−/−^ mice exhibit only partial novel object recognition deficits, it could suggest that hippocampus-mPFC projections remain intact. However, when performing the T-maze task to assess learning and working memory, PSD-95^−/−^ mice could not reach criterion (70% correct responses) even after 14 days compared to control mice beginning at day 6, indicating severe learning deficits. Not surprisingly, PSD-95^−/−^ mice performed significantly worse during delay tasks at 5 sec, 15 sec, and 60 sec (≤55% correct responses), suggesting working memory impairments. No significant differences were observed at 30 sec, and is likely attributed to the slight decline from control mice (63.89%). We associate these learning deficits with our electrophysiological data, more specifically, an attenuation in AMPAR-eEPSPs following a 10-pulse stimulation, further illustrating the effects of a reduction in GluA1 protein expression in the mPFC of PSD-95^−/−^ mice. Together, these results suggest that PSD-95 deficiency severely impairs mPFC-associated behavior that includes deficits in sociability, learning, and cognition. However, a discord of interpreting these results is the utilization of a whole-brain PSD-95 knock-out model rather than an mPFC-specific PSD-95 knockout. For instance, other regions containing mPFC projections such as the ventral hippocampus (memory consolidation and retrieval), basolateral amygdala (emotional control), and mediodorsal thalamus (working memory), may severely influence behavioral attributes of PSD-95^−/−^ mice^47,48^. Therefore, utilizing optogenetics techniques to understand the circuits involved would likely further elucidate the behavioral phenotypes of PSD-95 deficient mice.

We conclude that PSD-95 deficiency disrupts NMDAR/AMPAR balance and attenuates glutamatergic transmission within the mPFC. Since NMDARs are important for synaptic plasticity and cortical development, and involved in learning and memory, PSD-95 deficiency would alter these processes due to an aberrant upregulation of NMDARs at the synapse. Additionally, a reduction in AMPARs at the synapse may delay synaptic plasticity, and thus will affect learning and memory. Altogether, our results revealed alterations at the molecular, physiological, and behavioral levels in PSD-95 deficient mice during the adolescent age range; however, whether these changes persist into adulthood have yet to be investigated. Based upon our data, that reveal a progressive increase in NMDAR/AMPAR ratio from P21-to-P35, we expect an exaggerated decrease in GluA1 and AMPAR function, accompanied with dramatic increases in GluN1 and GluN2B and NMDAR function in adult (>postnatal day 70) PSD-95 deficient mice, thereby exacerbating behavioral phenotypes. Additionally, in *Dlg4*^−/−^ mice, cognitive deficits were observed during adulthood, therefore indicate persistent physiological aberrations^38^. Furthermore, examining spine density within layer 5 pyramidal neurons would be critical for understanding the structural effects of PSD-95 deficiency. Acquiring this data would be important for applying treatments such as memantine (an NMDAR antagonist) or LY395756 (mGluR2 agonist and mGluR3 antagonist), which were shown to balance NMDAR/AMPAR expression and function indirectly, and could rescue mPFC-associated behavioral deficits^49,50^. Thus, the data from our study may provide future therapeutic options that alleviate glutamatergic dysfunction in response to PSD-95 deficiency.

## Materials and Methods

### Animals

To model PSD-95 deficiency, we acquired PSD-95 knock-out mice from Jackson laboratories (B6.129-*Dlg4*^*tm1Rlh*^/J). Standard breeding procedures were used to generate homozygous PSD-95^−/−^ mice. All mice were genotyped using PCR techniques described in the Supplemental information (Supplementary Fig. 1). Both male and female C57/BL6 mice were used for all experiments and were divided into juvenile (Postnatal days, P14-21) and adolescent (Postnatal days, P35-55) groups as reported in prior studies^51–53^. The mice were cared for according to the National Institutes of Health (NIH) guidelines. Our animal experiment protocol was approved by the Institute Animal Care and Use Committee (IACUC) at Drexel University College of Medicine.

### Tissue collection and synaptosomal protein preparation

Mice aged P21 and P35 were anesthetized with euthasol (0.2 mg/kg, i.p.). Once unresponsive to toe- and tail-pinch, mice were transcardially perfused with ice-cold perfusion buffer. The medial prefrontal cortex was micro-dissected and isolated from mice and homogenized in a sucrose buffer (320 mM sucrose, 4 mM HEPES‐NaOH buffer, pH 7.4, 2 mM EGTA, 1 mM Na_3_VO_4_, 0.1 mM PMSF, 50 mM NaF, 10 mM Na_4_P_2_O_7_, 20 mM C_3_H_9_O_6_P, 1 μg/mL leupeptin, and 1 μg/mL aprotinin). Homogenates were centrifuged at 1,000 g for 10 min at 4 °C to remove nuclear materials and large cell fragments. The supernatant was centrifuged at 15,000 g for 15 min to yield cytoplasmic proteins and the pellet was hypo‐osmotically lysed and re‐suspended in homogenization buffer. The suspension was incubated for 30 minutes at 4 °C with continuous mixing, and then centrifuged at 25,000 g for 30 min to isolate synaptosomal protein fractions. Protein concentrations were measured using the Magellan protein assay machine (Tecan). Protein samples were made with lysis buffer, Laemlli sample buffer and b-mercaptoethanol to a total volume of 10 µg of protein.

### Western Blot and co-immunoprecipitation

The western blot and co-immunoprecipitation procedure were performed as previously described^54,55^. The mPFC protein samples were boiled at 95 °C for 5 min and then loaded into an SDS–PAGE gel for electrophoresis. Following electrophoresis, gels were transferred to polyvinylidene difluoride (PVDF) membranes (Millipore Billerica, MA) for 1 hr at 100 V, 4 °C. Membranes were blocked with 5% non-fat dry milk in TBST (0.05% Tween-20 in 1X Tris-buffered saline) for 1 hr and incubated in the following dilutions of primary antibodies for 1 hr: mouse monoclonal anti-GluN1 (1:2000, ThermoFisher Scientific Cat# 32-0500, RRID: AB_2533060), rabbit monoclonal anti-GluN2A (1:1000, Millipore Cat# 04-901, RRID: AB_1163481), mouse monoclonal anti-GluN2B (1:1000, Millipore Cat# 05-920, RRID: AB_417391), rabbit polyclonal anti-GluN3A (1:1000, Millipore Cat# 07-356, RRID:AB_2112620), mouse monoclonal anti-GluA1 N-terminus (1:1000, Millipore Cat# MAB2263, RRID: AB_11212678), mouse monoclonal anti-GluA2 (1:1000, Millipore Cat# MABN71, RRID: AB_10806492), rabbit polyclonal anti-PSD-95 (1:5000, Millipore Cat# AB9708, RRID:AB_2092543), rabbit polyclonal anti-SAP-102 (1:1000, ThermoFisher Scientific Cat# PA5-29116, RRID:AB_2546592), and mouse monoclonal anti-PSD-93 (1:1000, Millipore Cat# MABN497). A monoclonal mouse anti-β-actin (1:20,000, Sigma-Aldrich Cat# A5316, RRID: AB_476743) primary antibody was used to generate control bands. Blots will be rinsed with TBST 3x 20 minutes, and membranes will be incubated in horseradish peroxidase (HRP)-conjugated goat anti-mouse or rabbit IgG (mouse, Vector Laboratories Cat# PI-2000, RRID: AB_2336177; rabbit, Cat# PI-1000, RRID: AB_2336198) at 1:10000 for 1 hr. Protein bands were detected with the ECL Western Blotting System (Amersham ECL Western Blotting Detection Reagent, RPN2106). Membranes were then exposed to films and band densities measured with *Image J* (NIH). Data were normalized to levels of β-actin.

The mPFC tissue protein samples were further used for co-immunoprecipitation assays. 25 µg/µl of synaptosomal proteins were incubated overnight with 2.5 µg of anti-SAP-102 or anti-PSD-93. The immunocomplexes were isolated with 100 µl of Protein G MagBeads (GenScript, Cat# L00274) and incubated for 1–2 h at room temperature. Immunoprecipitates were washed three times with 1x phosphate buffered saline (PBS) and resuspended in Laemlli sample buffer, and boiled for 5 min. The supernatant was collected and immunoprecipitates were identified using standard western blot procedure as described above. Antibodies were directed against GluN2B, and against GluA1 as a negative control. Samples from each animal were run at least 3 times to minimize interblot variance. Results are presented as mean ± SEM and significance determined with Student’s *t*-test.

### Whole-cell patch clamp recordings

Whole-cell patch clamp techniques were used to record from layer V pyramidal neurons from coronal brain slices of the mPFC as previously described^21,56,57^. Mice aged P21-25 and P35-39 were deeply anesthetized with Euthasol (0.05 ml, _I.P._) and decapitated. Brains were quickly removed and placed in an ice-cold sucrose solution (in mM): 87 NaCl, 75 sucrose, 2.5 KCl, 2 CaCl_2_, 7 MgCl_2_, 1.25 NaH_2_PO_4_, 26 NaHCO_3_, and 25 dextrose. Coronal brain slices of the mPFC were made at 300 µm thickness using the Leica VT1200 S Vibratome. Slices were then transferred to an oxygenated artificial cerebrospinal fluid (ACSF) solution (in mM): 124 NaCl, 2.5 KCl, 2 CaCl_2_, 1 MgCl_2_, 1.25 NaH_2_PO_4_, 26 NaHCO_3_, and 10 dextrose, pH 7.4 and incubated at 36 °C for 40 min. Individual slices were transferred to a recording chamber continuously perfused with ACSF kept at 36 °C.

#### Evoked excitatory postsynaptic currents (eEPSCs)

Whole-cell voltage clamp techniques were used to record NMDAR and AMPAR eEPSCs in the mPFC. An upright microscope (Olympus BX51WI, Olympus Optics, Japan) equipped with infrared-differential interference contrast (IR-DIC) optics was used for recordings. Patch electrodes were filled with Cs^+^-containing solution (in mM): 120 Cs-gluconate, 5 lidocaine, 6 CaCl_2_, 1 Na_2_ATP, 0.3 Na_2_GTP, and 10 HEPES (pH 7.3 adjusted by CsOH). To measure NMDAR- and AMPAR-mediated current, a bipolar electrode was used to stimulate layer II/III and record layer V pyramidal neurons in the mPFC. To stimulate, a single pulse was elicited (0.1 ms, 10–100 µA, 0.1 Hz). Picrotoxin (PTX; 50 mM, Sigma-Aldrich, St. Louis, MO, Cat# 124-87-8) was applied to block GABAergic transmission. Isolated NMDAR-mediated currents were recorded at +60 mV under a bath application of both picrotoxin and AMPAR antagonist CNQX (20 µM, Sigma-Aldrich, Cat# 115066-14-3). GluN2B-NMDAR currents were blocked with a selective GluN2B antagonist, Ro25-6981 (Ro25, 1 µM, Sigma-Aldrich, MO, Cat# 1594/1).

#### Miniature excitatory postsynaptic currents (mEPSCs)

To isolate miniature AMPA receptor currents, the membrane potential was held at −70 mV with Cs^+^-containing solution intracellular solution in the presence of picrotoxin and tetrodotoxin (TTX, 0.5 µM, Hello Bio, Princeton, NJ, Cat# HB1034). The mEPSCs from layer V pyramidal neurons were recorded for 5 min, and the frequency and amplitudes were measured by averaging 5 sweeps from the on-set of recording.

*10-pulse temporal summation of evoked excitatory postsynaptic potentials* (*eEPSPs*). Whole-cell current clamp recordings were used to measure eEPSPs. Patch electrodes were filled with potassium gluconate internal solution and a bipolar electrode was used to stimulate layer II/III and record from layer V pyramidal neurons in the mPFC as similarly described above. A train of 10 pulses/stimuli were delivered at 20 Hz to record eEPSPs. The membrane potentials of the recording cells were adjusted within −68 mV to −73 mV through a small holding current.

*Data analysis*. Electrophysiological experiments were conducted with the Axon MultiClamp 700B amplifier (Molecular Devices). Data acquisition was from pCLAMP 9.2 software and analyzed using Clampfit 9.2 (Molecular Devices). The eEPSCs amplitudes were measured by averaging 30 sweeps from the onset to the peak amplitude of the EPSCs. The NMDAR/AMPAR-mediated current ratio was calculated by measuring AMPAR peak value at −60 mV and NMDAR peak value at +60 mV 50-ms post-stimulus that is illustrated by a yellow circle on EPSC trace examples. The kinetics of NMDAR-EPSC decay (τ) were measured by standard exponential fitting 63% of the EPSC peak amplitude and is represented in milliseconds (ms). To analyze sEPSCs, we select a sample sEPSC as a template within the 5 min data acquisition period. The frequency (number of events/300 = Hertz) and amplitude (peak) of the individual events were examined with a threshold in Clampfit. Neurons that produced stable baseline EPSCs were used for analysis. EPSC data were analyzed with the two-tailed Student *t-*test for statistical significance and were presented as mean ± S.E.M. The 10-pulse AMPAR-eEPSPs were recorded in current clamp mode, and peak voltages were measured and normalized to calculate temporal summation. Repeated-measures analysis of variance (ANOVA) with paired *t*-test was used to determine significance. The paired-pulse ratio was determined as the peak voltage of EPSP2/EPSP1.

### Locomotor activity

Locomotor activity was conducted on mice aged P35 in an open field over a 30 min period using a novel 43.2 cm × 43.2 cm activity test chamber with infrared beams (Med Associates, Inc., St. Albans, VT, USA). Within the open-field chamber, horizontal counts (number of invisible infrared beam breaks) were automatically recorded for the duration of 30 min. Repeated measures of ANOVA followed by Bonferroni’s test were used for statistical analysis.

### Sociability tasks

The sociability test was performed on mice aged P35 within a 3-chamber apparatus (box measures 62 × 43 × 20 cm; individual 3 chambers 19.5 × 43 cm). The tasks were divided into two sessions assessing social approach and social exploration/novelty^58,59^; and is further described in the Supplemental information. Both sessions were 10 min, and the time in each chamber and sniffing time (nose pokes) were measured. ANOVA followed up with a paired *t-*test were used for statistical analysis. p < 0.05 was considered statistically significant.

### Cognition tasks

First, to evaluate recognition memory, a novel object recognition task was performed on mice aged P35. Object sniffing time was measured between the familiar object and novel object, and discrimination ratio (novel object interaction/total interaction with both objects) was calculated to score object recognition. A one-sample *t*-test was used to compare individual groups to the discrimination ratio of 0.5 (50%). To compare the groups a two-tailed Student *t-*test was for statistical significance and presented as mean ± S.E.M. p < 0.05 was considered statistically significant. Next, to evaluate learning and working memory, a discrete paired-trial delayed alternation training task using a T-maze apparatus (50 × 72 cm) was used as previously reported^60,61^. Mice were required to reach criterion (70% correct) to begin testing, which consisted of variable intra-trial delays of 5 s, 15 s, 30 s, and 60 s. Repeated measures of ANOVA with Tukey-Kramer *post hoc* test were used for statistical analysis of the working memory task. Details of novel object recognition and T-maze working memory tasks are in the Supplemental information.

## Supplementary information

Coley_Gao_Supplemental Info
