# Supplementary material for: PSD-95 deficiency disrupts PFC-associated function and behavior during neurodevelopment
Source: Sci Rep. 2019 Jul 1;9:9486. doi: 10.1038/s41598-019-45971-w (PMC6602948; doi:10.1038/s41598-019-45971-w)
Supplement: Supplementary file 1 — Coley_Gao_Supplemental Info [file 41598_2019_45971_MOESM1_ESM.docx]

PSD-95 deficiency disrupts PFC-associated function and behavior during neurodevelopment

Austin A. Coley and Wen-Jun Gao

**Supplementary Materials and Methods**

***Animal***

PSD-95 knock-out mice (PSD-95^-/-^) were acquired from Jackson laboratories (B6.129-*Dlg4^tm1Rlh^*/J)(Stock number: 013099). The Richard Huganir laboratory (Johns Hopkins University) designed the PSD-95 knock-out mouse model via homologous recombination techniques A targeted construct containing neomycin cassette was used to replace exons containing PDZ 1 and 2 domains of PSD-95 to generate an out-of-frame transcript. This targeted mutation disrupted PSD-95 protein expression, and thus considered a knock-out. All animal subjects, male and female C57/BL6 mice aged postnatal days P21-P55, PSD-95^-/-^ mice are used as experimental mice and littermates PSD-95^+/+^ mice are used as controls. Standard breeding procedures were used to generate homozygous PSD-95^-/-^ mice. All mice were genotyped using PCR techniques. The primers used for *Dlg4^tm1Rlh^* PCR assay were DLG4 WT-FWD (5’- AAA CCC AGG AGC AGA GGTTTC ATG ACA -3’), DLG4 WT-REV (5’- TCA TAG GGG TCC ATC AGT CTC TGG CT -3’) and DLG4 Mutant (5’- ATG CTC CAG ACT GCC TTG GGA AAA G -3’).

**Action Potentials recordings**

To record action potentials, whole-cell current clamp was used and patch electrodes were filled with potassium gluconate internal solution (in mM): 120 potassium gluconate, 20 KCl, 4 ATP-Na, 0.3 Na_2_GTP, 5 Na-phosphocreatine, 0.1 EGTA, 10 HEPES, pH 7.3, 305 mosmol/l). Action potential responses were measured in response to various step currents from -300 pA to +400 pA with 50 pA increments. The resting membrane potential, input resistance, action potential (AP) threshold, AP half-width, and peak AP amplitude were also measured.

Action potentials were recorded in current clamp mode, and spike numbers in response to depolarizing current injections were measured. Repeated-measures ANOVA with paired *t*-test was used to determine significance. p<0.05 was considered statistically significant.

***Behavioral analysis***

*3-chamber sociability.* The sociability test was performed using a 3-chamber apparatus (box measures 62 x 43 x 20 cm; individual 3 chambers 19.5 x 43 cm). In the first session, a subject mouse is placed inside the center chamber of the apparatus and interacts with either a novel mouse inside an enclosure in a side chamber, or a novel object/enclosure in the opposite side chamber. In the second session, the subject mouse is placed in the center chamber and interacts with either a familiar mouse inside an enclosure in the side chamber, or an intruder mouse inside an enclosure in the opposite side chamber. Both sessions were 10 min, and the time in each chamber and sniffing time were measured. ANOVA followed up with paired *t-*test were used for statistical analysis. p<0.05 was considered statistically significant.

*T-maze working memory.* To assess working memory, a discrete paired-trial delayed alternation training task was used. Mice were on a food-restricted diet consisting of ~2.0 g of food per day to maintain 85% of their baseline body weight. Mice were given 2 days of habituation to the T-maze apparatus (50 x 72 cm), and then underwent discrete paired–trial delayed alternation training, where each trail consisted of a forced run with a blocked arm and a choice run with both arms open. To reach criterion, mice were required to achieve 70% correct trials in 3 consecutive days. A maximum of 14 days was allowed for each mouse to reach criterion. Mice then underwent 3 days of testing that consists of a random sequence of 16 trials each day and variable intra-trial delay of 5 s, 15 s, 30 s, and 60 s. Repeated measures of ANOVA with Tukey-Kramer *post hoc* test were used for statistical analysis of the working memory task.

*Novel object recognition.* To evaluate recognition memory, a novel object recognition task was utilized. Mice were habituated in an open-field box without objects (29 x 29 cm) for 5 min. On the following day when mice were placed in the box with two identical objects and allowed to explore for 5 minutes. After 1 hr, one of the objects was replaced with a novel object, and the subject mouse was placed in the box for 5 min for the testing session. Object sniffing time was measured between the familiar object and novel object, and discrimination ratio (novel object interaction/total interaction with both objects) was calculated to score object recognition. The two-tailed Student *t-*test was for statistical significance and presented as mean ± S.E.M. p<0.05 was considered statistically significant.

**Supplementary Figures**


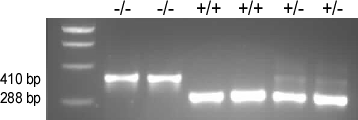


**Supplementary Figure 1.** PSD-95 genotyping in wild-type control, heterozygous, and homozygous mice. PCR assay shows duplicates of PSD-95^-/-^ (410 bp), wild-type PSD-95^+/+^ (288 bp), and PSD-95^+/-^ (410 and 288 bp).

**
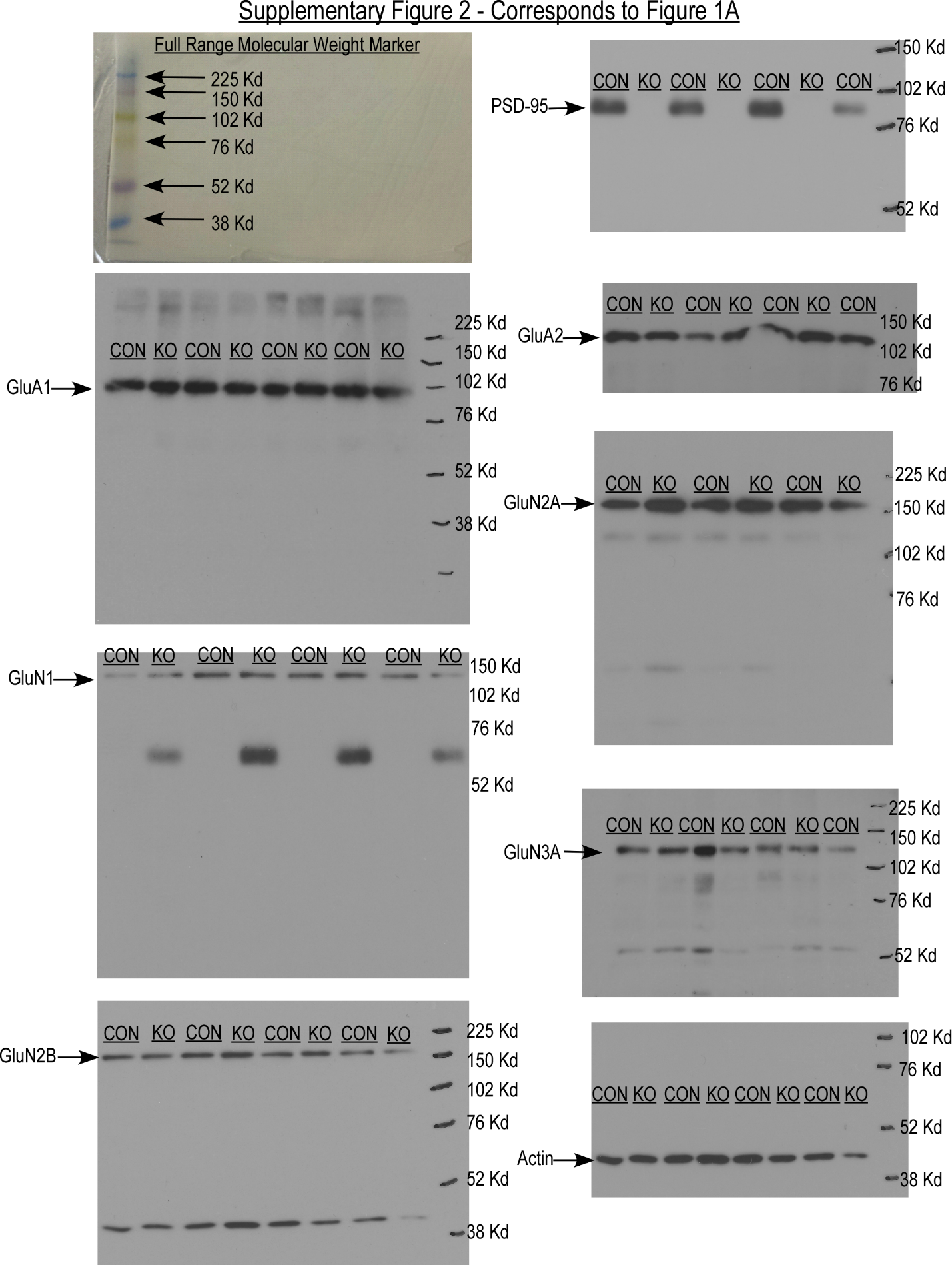
**

**
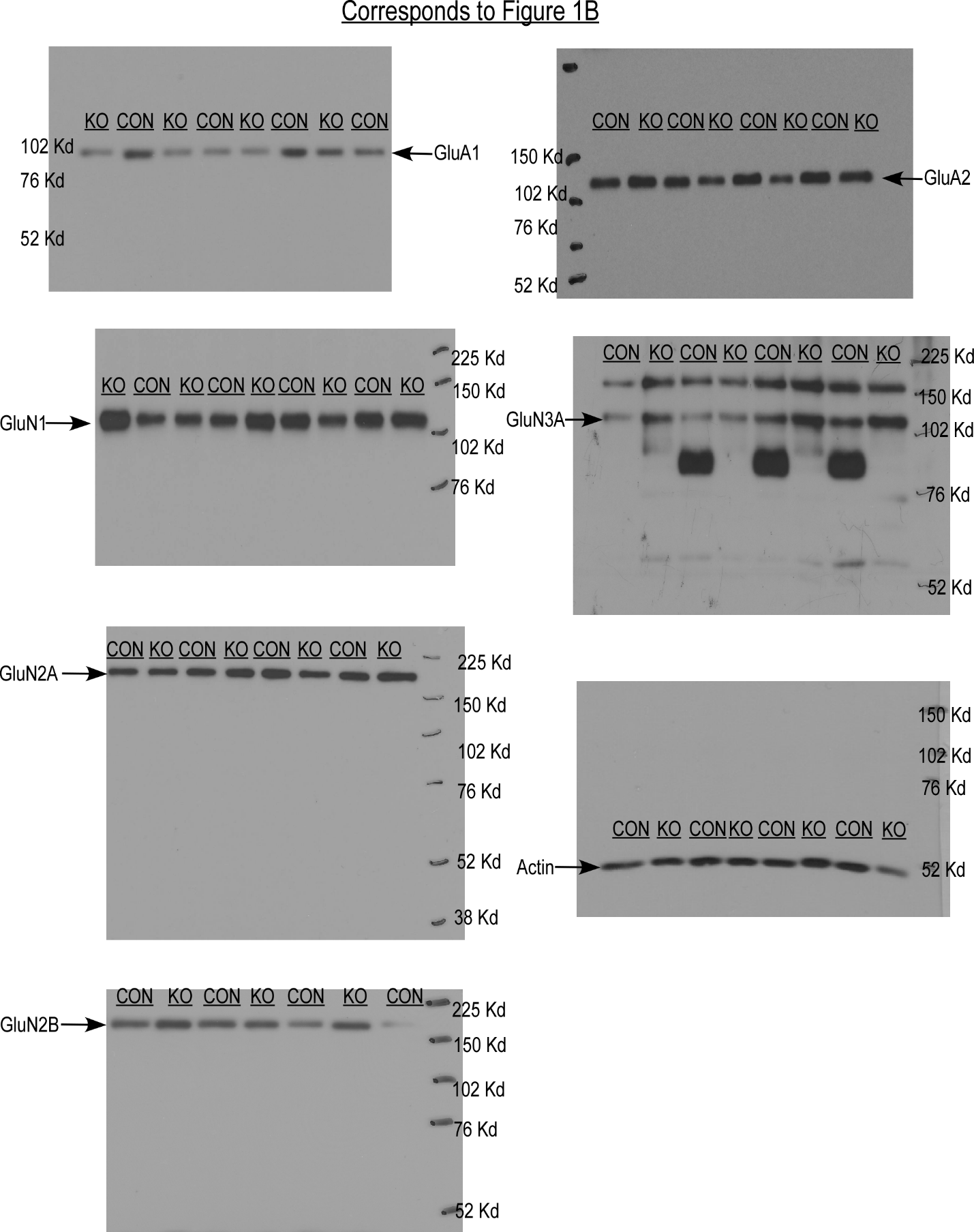
**

**
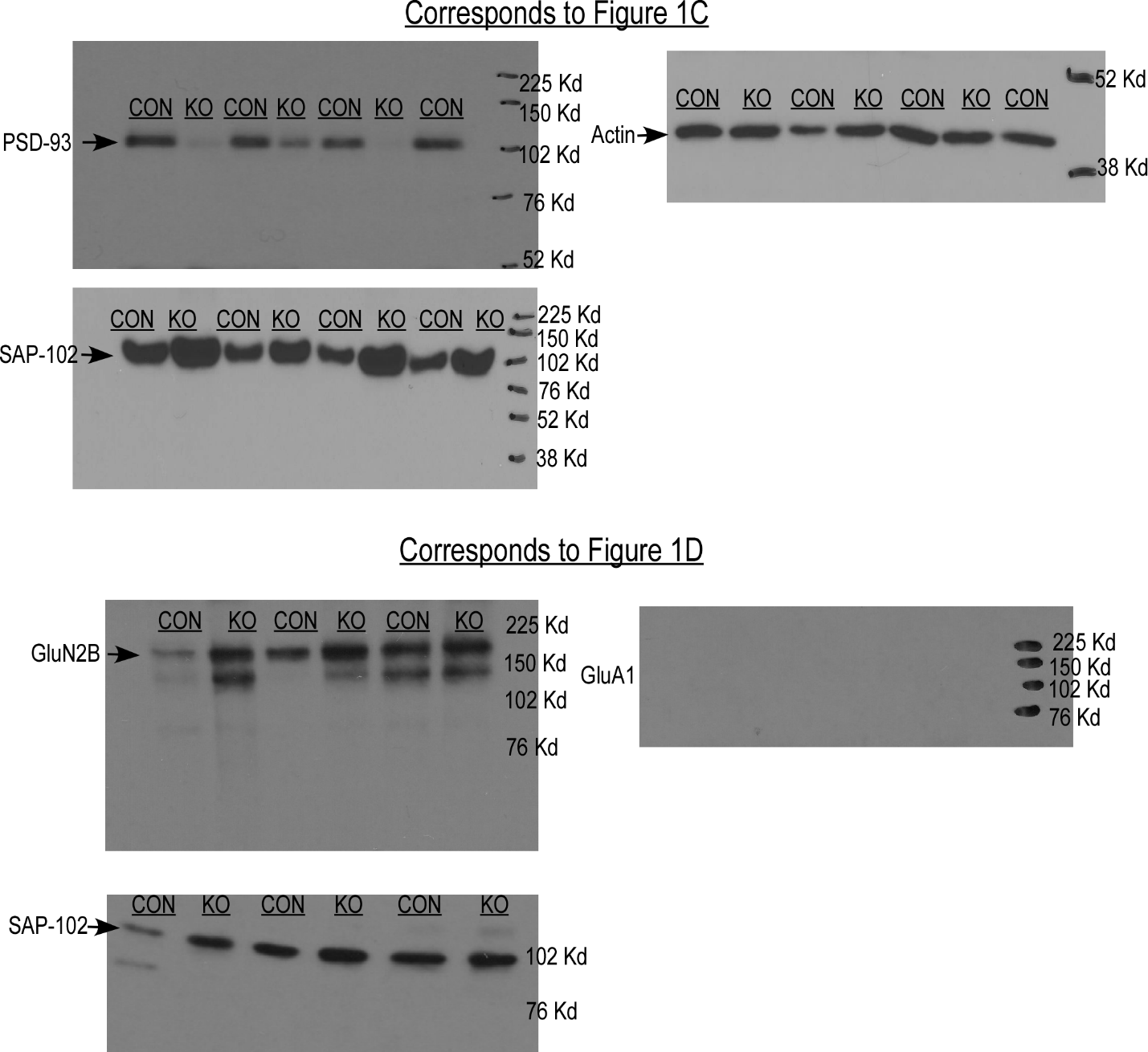
**

**Supplementary Figure 2.** Uncropped representative western blots for PSD-95 and corresponding figures 1A-1D describing AMPAR subunits (GluA1 & GluA2), NMDAR subunits (GluN1, GluN2A, GluN2B, and GluN3A), scaffolding proteins (SAP-102 & PSD-93), and actin in Con vs. KO (PSD-95^-/-^) mice at P21 and P35 time points.

**
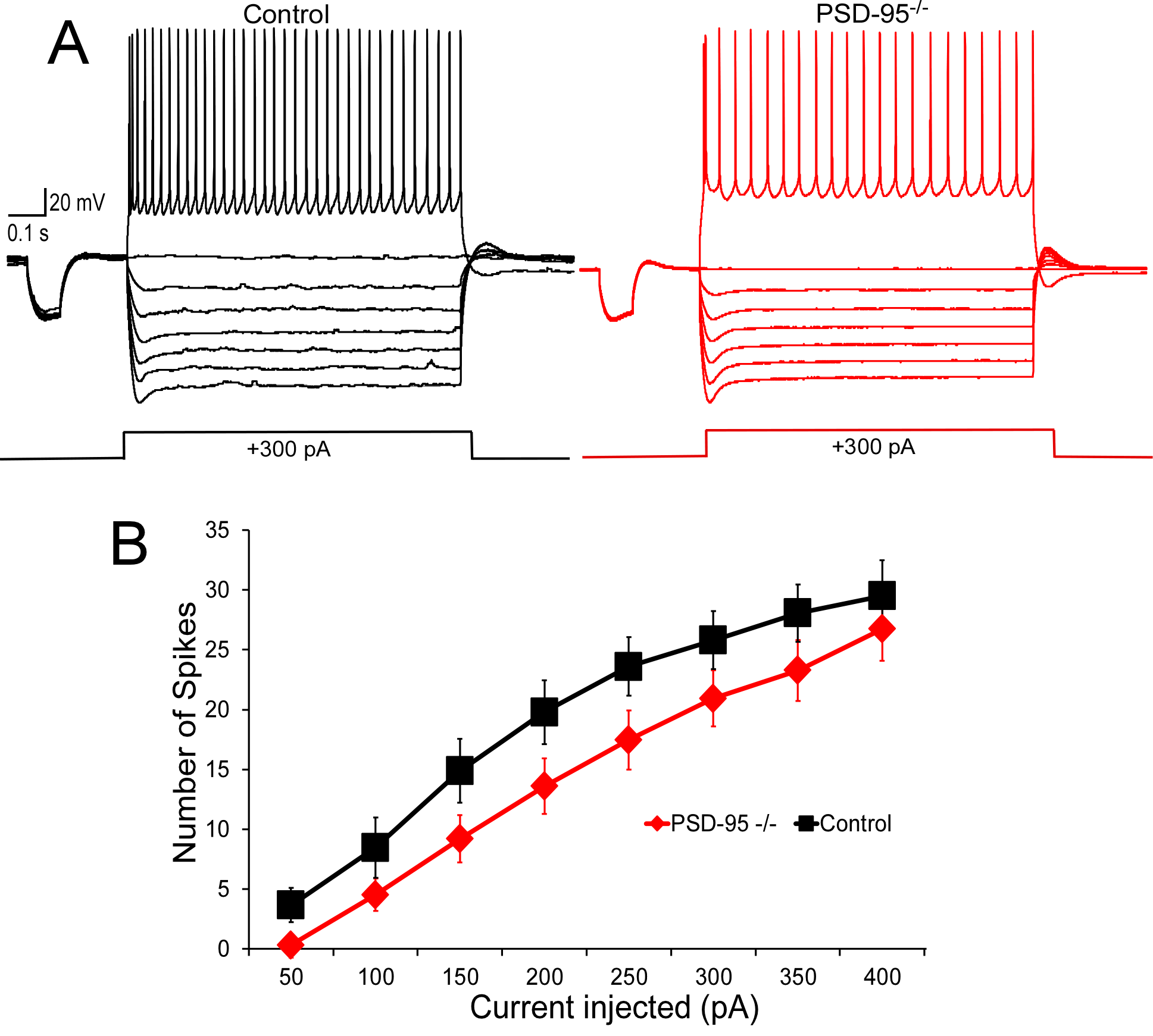
**

**Supplementary Figure 3.** (A) Example traces of action potentials from layer V pyramidal neurons in Con vs. KO mice. (B) Line graph displays relationship between action potential spike numbers (y-axis) vs. current injection (x-axis) in Con vs. KO mice (repeated measures of ANOVA, F=(1,28)=2.47, p=0.13, n=15).


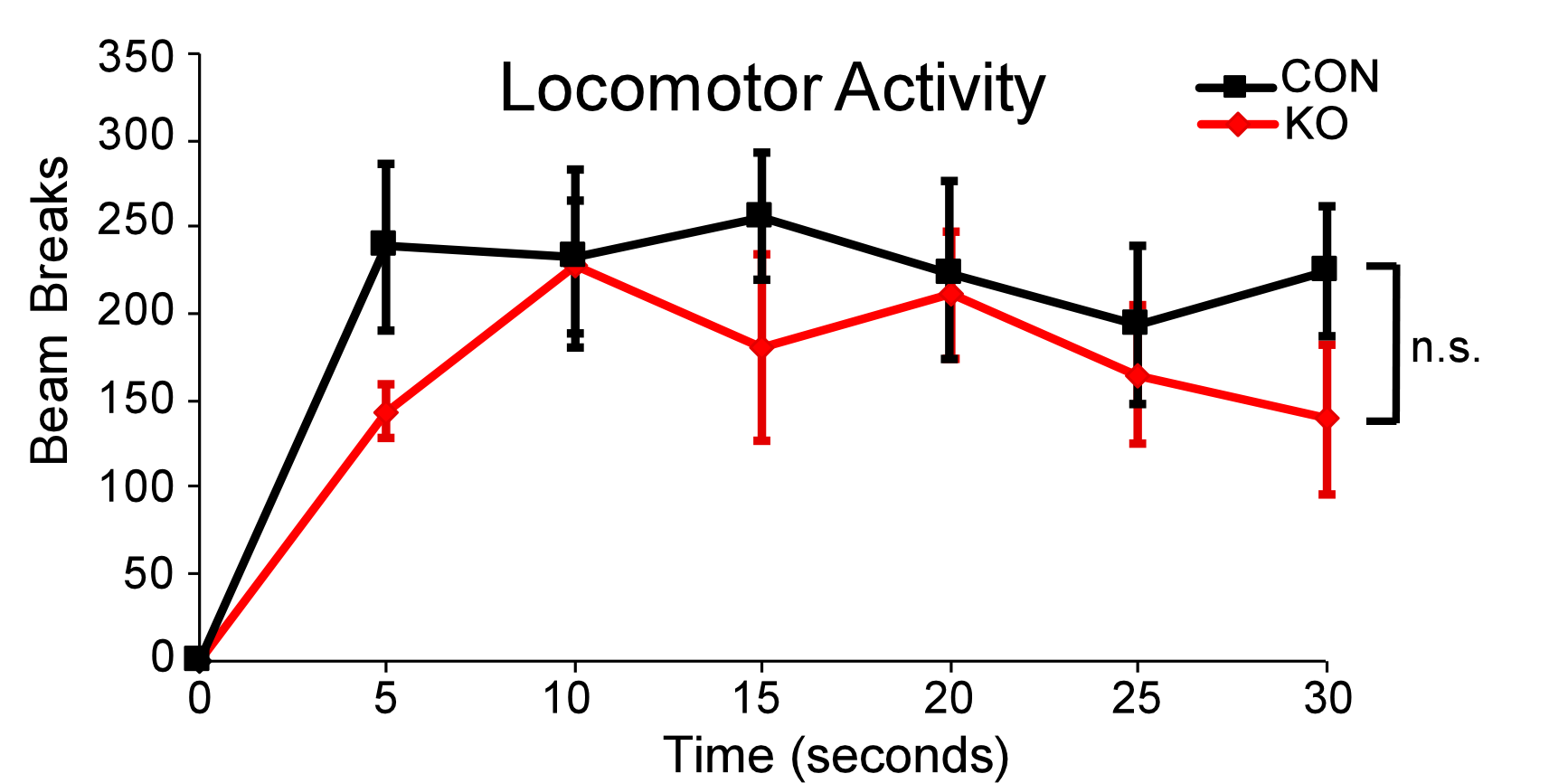


**Supplementary Figure 4.** Line graph represents the relationship between beam breaks (y-axis) vs. time in seconds (x-axis). No significant differences in spontaneous locomotion activity in Con vs. KO mice (repeated measures of ANOVA, F=(1,9)=0.900, p=0.37, CON n=6, KO, n=5). n.s., not significant.

**Supplementary Table 1.** Physiological properties of layer 5 pyramidal neurons in PSD-95^-/-^ mice

|  | **Resting membrane potential (mV)** | **Input resistance (MΩ)** | **Rheobase (pA)** | **Action potential threshold (mV)** | **Action potential peak amplitude (mV)** | **Action potential ½ width (ms)** |
| --- | --- | --- | --- | --- | --- | --- |
| Control mice | -67.37 ± 2.57 | 159.53 ± 13.59 | 90 ± 12.15 | -29.94 ± 1.86 | 59.52 ± 2.99 | 1.39 ± 0.08 |
| PSD-95^-/-^ mice | -68.04 ± 1.36 | 129.32 ± 13.35 | ** 143.33 ± 14.53 | -31.59 ± 1.9 | 59.3 ± 2.25 | 1.21 ± 0.10 |

There is a significant increase in the Rheobase in layer V pyramidal neurons in PSD-95^-/-^ mice (**p<0.01). There were no significant differences in resting membrane potential, input resistance, action potential threshold, action potential peak amplitude, and action potential ½ width in layer V pyramidal neurons in PSD-95^-/-^ mice compared to control mice (p>0.05).
